# A 150 kDa Protein Derived from Bull Seminal Plasma Extended the Survival Time of Kacang Goat Sperm Stored at 5°C

**DOI:** 10.1155/2021/1470209

**Published:** 2021-11-18

**Authors:** Suherni Susilowati, Imam Mustofa, Wurlina Wurlina, Indah Norma Triana, Suzanita Utama, Budi Utomo

**Affiliations:** Division of Veterinary Reproduction, Faculty of Veterinary Medicine, Universitas Airlangga, Kampus C Unair, Mulyorejo, Surabaya 60115, Indonesia

## Abstract

Artificial insemination has proven to be an effective method for increasing population size and genetic quality of Kacang goats. However, innovation is required to maintain the quality of Kacang goat semen in storage. This study aimed to examine the effects of supplementing the 150 kDa protein assumed as IGF-I complex derived from bull seminal plasma in skim milk-egg yolk extender on the quality of Kacang goat sperm stored at 5°C. Twelve ejaculates collected from three Kacang goats were divided into three groups. In the control group (T0), the ejaculates were extended with skim milk-egg yolk only. In the treatment groups (T1 and T2), the ejaculates were extended with skim milk-egg yolk supplemented with the IGF-I complex protein at 12 *μ*g and 24 *μ*g/100 mL, respectively. The extended semen was stored at 5°C, and the viability, motility, intactness of the plasma membrane, malondialdehyde concentration, and apoptotic sperm percentage were evaluated daily for five days. The results showed that the T1 was the most effective treatment for maintaining Kacang goat semen at a quality acceptable for artificial insemination over five days of storage at 5°C. However, the T0 and T2 groups retained acceptable qualities for only three days at 5°C. It could be concluded that supplementation of 12 *μ*g of the 150 kDa protein derived from bull seminal plasma per 100 mL extender successfully extended the life span of Kacang goat sperm for five days.

## 1. Introduction

The Kacang goat is a small ruminant that is reared by many people in rural areas of Indonesia. This livestock commodity provides a source of additional income to prevent poverty and animal protein to improve nutrition. However, the development and genetic quality of the population are unsatisfactory. To address these problems, semen can be obtained from superior male Kacang goats for artificial insemination. Unfortunately, frozen Kacang goat semen is not viable, but fresh semen can be diluted in an extender and stored at 5°C to increase the survival time. Survival time is defined as the period from the initial qualification to when the minimum prerequisite sperm motility limit for artificial insemination is reached, based on established standards [1].

At tropical room temperature (∼23°C), Kacang goat fresh sperm survival time is only 15 h [2]. Sperm metabolic oxidation results in the production of lactic acid as a waste product, which subsequently causes a rapid decrease in sperm motility. High concentrations of lactic acid damage the plasma membrane through lipid peroxidation [3]. Storage of semen at 5°C is useful for maintaining survival motility for several days [4], enabling longer transport times [5]. Sperm metabolism is slowed at the chilling temperature, which reduces the production of lactic acid and extends the life span of the sperm. However, Kacang goat sperm is susceptible to cold temperature stress. The previous study showed that diluting fresh goat semen resulted in a significant decrease in progressive motility from 85% to the limit of 40% in four days of storage at 5°C [6]; meanwhile, Kacang goat semen reached the 40% motility limit from an initial 90% in only three days [7]. Cold stress from chilling at 5°C resulted in deterioration of the plasma membrane, followed by decreased motility and viability of Ettawa goat sperm [6].

The susceptibility of goat sperm to cold temperature stress is mediated by lipid peroxidation [8] of the high levels of unsaturated fatty acids in the sperm membrane [9]. Lipid peroxidation results in the production of reactive oxygen species (ROS) and malondialdehyde (MDA), causing damage to the plasma membrane, acrosome, and sperm morphology [10]. However, antioxidants can play a role in binding free radicals to prevent oxidative damage [11, 12].

The previous study showed that the addition of Simmental bull whole seminal plasma to goat sperm improved postthaw sperm quality [13, 14]. The seminal plasma contains a 150 kDa ternary complex consisting of an IGF, insulin-like growth factor binding protein-3 (IGFBP-3) (or less often IGFBP-5), and a glycoprotein called acid-labile subunit [15]. Insulin-like growth factor-1 (IGF-I) is secreted by Leydig and Sertoli cells in the seminal plasma. It is presumed that the protective function of IGF-I is due to its antioxidant activities [16]. Therefore, this study aimed to examine the effects of a 150 kDa protein derived from Simmental bull seminal plasma (hereinafter referred to as “the protein”) on the survival, viability, motility, plasma membrane, apoptosis, and malondialdehyde concentration of goat sperm stored at 5°C.

## 2. Material and Methods

### 2.1. Ethical Approval

The study was conducted based on the approval of the Animal Care and Use Committee, Airlangga University, Surabaya, Indonesia, no. 520/HRECC.FODM/VII/2019. The committee assessed the proposal of this study based on animal welfare principles, the UK Animals (Scientific Procedures) Act, 1986, and associated guidelines, EU Directive 2010/63/EU for animal experiments. The collection of bull and goat semen was conducted according to the protocol of Chapter 4.7 (the collection and processing of bovine, small ruminant, and porcine semen) of the Terrestrial Animal Health Code of the World Organization for Animal Health.

### 2.2. Experimental Animals

Two elite Simmental bulls and three male Kacang goats were reared at the Teaching Farm of the Faculty of Veterinary Medicine, Airlangga University, Surabaya. Semen is routinely collected from these Simmental bulls (age: 5-6 years; weight: 400–900 kg) for frozen semen production at the Regional Artificial Insemination Center of Universitas Airlangga. The healthy male Kacang goats were 3–5 years old, weighing 25–35 kg, and had high libidos.

### 2.3. Semen Collection

Simmental bull and Kacang goat were collected twice a week using an artificial vagina. The fresh semen was examined macroscopically (volume, odor, pH, consistency, and color) and microscopically (motility, viability, and concentration). The sperm used in the study had at least 70% motility and viability [1].

### 2.4. Purification of Bull Seminal Plasma Protein

Four Simmental bull ejaculates were used for the study. Each ejaculate was combined with phosphate buffer saline (PBS) and centrifuged at 1,800 rpm for 10 min at 5°C, and then the supernatant (semen plasma) was collected using a micropipette. To purify the seminal plasma proteins, PBS and phenylmethanesulfonyl fluoride (PMSF) were added, and the mixture was vortexed for 5 min, sonicated for 10 min at 4°C, then vortexed again, and centrifuged at 6,000 rpm for 10 min. The supernatant was combined with absolute ethanol in a ratio of 1 : 1 and allowed to precipitate overnight. The ethanol was removed, and the resulting pellet was combined with Tris-HCl at 1-2 times the pellet volume [17].

### 2.5. Identification of IGF-I Complex

Identification of IGF-I complex was using sodium dodecyl sulfate-polyacrylamide gel electrophoresis (SDS-PAGE) with 12.5% separating gel, 3% stacking gel, Broad Range (10–200 kDa) Protein Molecular Weight Markers (Bio_Rad Laboratories, Ivry-sur-Seine, France), and staining with Coomassie Brilliant Blue [17]. The percentage of IGF-I complex fraction in Simmental bull seminal plasma was measured by using Gel Quant Express 4.1 application for analysis of 1D gels (Microsoft™ Windows™).

Western blotting with protein molecular weight markers (range 4.4–200 kDa, Bio_Rad) was used to detect the specificity of the IGF-I complex derived from Simmental bull seminal plasma. The seminal plasma samples were diluted in SDS-PAGE sample buffer (50 mM Tris-HCl, pH 6.8, 2% SDS, 10% glycerol, 5% b-mercaptoethanol, and 0.1% bromophenol blue), heated 7 min at 95°C, and resolved on 7.5% SDS polyacrylamide gels [18]. The proteins were transferred to a 0.2 mm nitrocellulose membrane, and then washed using 0.1% phosphate buffer saline-tween (PBST) 5 minutes three times [19]. Membrane blocking process using 0.2% BSA at room temperature for 60 minutes, followed by washing with 0.1% PBST for 5 minutes three times. The primary antibody solution (IGF-1 monoclonal antibody, Sigma-Aldrich) 1 *μ*g/mL was added to the membrane and then incubated at 4°C overnight. After that, the membranes were washed using 0.1% PBST for 5 minutes three times. The next step was adding a secondary anti-IGF1 antibody solution (Goat anti-Bovine Insulin-Like Growth Factor I, Bio_Rad) 1 : 10,000 and then incubating at room temperature for 1.5 hours [20]. The membrane was rewashed using 0.1% PBST for 5 minutes three times, and the chemiluminescence substrate was added to the membrane, incubated for 5 minutes, and then detected the IGF-1 complex protein using a C-Digit LICOR [21].

### 2.6. Isolation of IGF-1 Complex Protein

The isolation of IGF-I complex protein from polyacrylamide gel was using a dialysis membrane for protein retention. The protein band with 150 kDa size of SDS-PAGE without staining was excised into small fragments. The gel fragments were equilibrated twice in 0.125 M Tris-HCl buffer (pH 6.8) and 2.0% of 2-mercaptoethanol for 15 min. A final equilibration of the gel fragments was conducted in 0.125 M Tris-HCl buffer (pH 6.8) 1.0% (w/v) SDS. The equilibrated gel fragments were then placed in a dialysis tube with a minimum amount of tris-glycine buffer containing SDS (25 mM Tris, 192 mM glycine, and 0.1% SDS). The electroelution was conducted in tris-glycine buffer containing 0.1% SDS (pH 8.3) at 50 V for 12 h at 4°C. The polarity of the electrodes was changed for one minute at the end of electrophoretic elution to avoid the absorption of protein on the dialysis tubes [22].

### 2.7. Measurement of IGF-I Complex Concentration

Spectrophotometric analysis was used to measure the concentration of IGF-1 complex isolate (IMV Photometer). A 0.02 mL sample of electrophoretic elution result was diluted in physiological solution (0.9% sodium chloride) 1 : 200 and placed in a cuvette. The result of the analysis was a print-out of the number of proteins [17, 23].

### 2.8. Extender Preparation and Chilled Storage

The skim milk-egg yolk (SM-EY) extender was prepared following the method described in a previous study [24]. In brief, 10 g of skim milk powder (Merck 115338) was dissolved in 100 mL of distilled water, heated to 92°C–95°C for 10 min, and then cooled to 37°C. The solution was added to 5 mL of homogenized egg yolk (derived from laboratory chicken eggs) to a total volume of 100 mL. Penicillin and streptomycin were added in concentrations of 1.0 IU/mL and 0.1 mg/mL, respectively [7]. The SM-EY extender was divided equally into three groups: control group (T0), with no additions; T1, with 12 *μ*g of the 150 kDa protein/100 mL extender added; T2, with 24 *μ*g of the 150 kDa protein/100 mL extender added. The dose of the 150 kDa protein added was based on our earlier study [25]. Six replicates were prepared for each treatment. The Kacang goat semen was diluted to a concentration of 300-million live sperm/mL before the extenders were added. The aliquots were stored at 5°C, and the quality was assessed daily for five days.

### 2.9. Semen Quality Assessment

Sperm viability, progressive motility, plasma membrane integrity, MDA level, and sperm apoptosis (%) were assessed according to the previously described [6].

### 2.10. Viability

A dry-smeared sample was stained with eosin-nigrosin, and 100 sperm were examined at 400× magnification under a light microscope (Olympus BX-53). The live sperm were identified by their bright transparent heads; dead sperm were identified by their reddish color [6].

### 2.11. Motility

A homogenized mixture of the semen sample and physiologic salt solution was covered on a glass object. The progressive motility of 100 sperm was assessed at 400× magnification under a light microscope (Olympus BX-53) on a heating table at 37°C–38°C [6].

### 2.12. Intact Plasma Membrane

Semen samples (0.1 mL) were diluted with 1 mL hypoosmotic solution and incubated at 37°C for 30 min. The plasma membranes of 100 sperm per sample were assessed under a light microscope (Olympus BX-53) at 400 × magnification. The hypoosmotic solution was a mixture of 1.352 grams fructose and 0.735 grams Na Citrate 2 H_2_O dissolved in 100 mL of distilled water. Intact plasma membranes were identified by circular tails; damaged plasma membranes were characterized by straight tails [6].

### 2.13. MDA Concentration

MDA concentration was measured using the thiobarbituric acid (TBA) method. The MDA kits (containing TBA concentrations of 0, 1, 2, 3, 4, 5, 6, 7, and 8 *μ*g/ml, resp.) and 100 *μ*l semen samples were dissolved in distilled water to 550 *μ*l before 100 *μ*l of 20% trichloroacetic acid was added and homogenized for 30 s. Next, 250 *μ*l of 1N HCl was added to the mixture and homogenized, and then 100 *μ*l of 1% sodium was added and homogenized before centrifuging at 500 rpm for 10 min. The supernatant was incubated in a water bath at 100°C for 30 min and then allowed to cool at room temperature. Absorption was measured at 533 nm using a spectrophotometer. The MDA concentration (ng/mL) was calculated by extrapolating the sample absorbance value to the standard MDA curve [6].

### 2.14. Apoptosis

Dry-smeared semen samples were fixed using absolute methanol and glacial acid for 15 min and then stained with acridine orange to examine the percentage of sperm apoptosis. For each sample, 100 sperm were examined under a fluorescence microscope at 100× magnification. Apoptotic sperm were characterized by a yellow to reddish appearance; normal sperm appeared green [6].

### 2.15. Data Analysis

Analysis of variance was used to detect the effects of the treatments on the percentage of motility, viability, intact plasma membrane (IPM), apoptosis, and MDA concentration, followed by Tukey's Honestly Significant Difference test at *p* ≤ 0.05 in SPSS (Statistical Product and Service Solutions, Version 23).

## 3. Results

**Figure 1:**
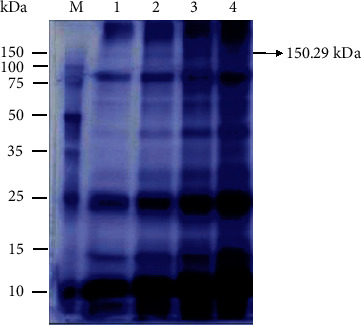
Sodium dodecyl sulfate-polyacrylamide gel electrophoresis image of protein bands for Simmental bull seminal plasma protein. kDa: kilo Dalton, M: marker, 1 and 2: seminal plasma of first Simmental bulls, and 3 and 4: seminal plasma of second Simmental bulls.

**Figure 2:**
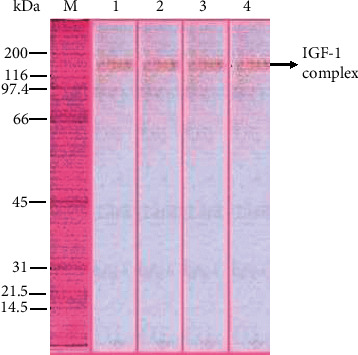
Western blot of IGF-1 complex protein derived from Simmental bull seminal plasma protein. kDa: kilo Dalton, M: marker, 1 and 2: seminal plasma of first Simmental bulls, and 3 and 4: seminal plasma of second Simmental bulls.

Six protein bands of the Simmental bulls seminal plasma were found (Figure 1; Table 1). The 150.29 ± 0.83 kDa presumed as IGF-I complex was verified in Western blotting using IGF-1 monoclonal antibody (Figure 2). The IGF-I complex (hereinafter referred to as “the protein”) obtained was 4.58 ± 0.16% of the total protein in the Simmental bulls seminal plasma with 1.18 ± 0.04 *μ*g/mL concentration (Table 1), and 65.12 *μ*g total of the protein was obtained.

The macroscopic characteristics of Simmental bull and Kacang goat ejaculates shared similarities. Both ejaculates had a thick consistency, yellowish-white color, a distinct smell, and pH around 7. However, some differences in the microscopic characteristics were observed between the two species. Substantial differences in the volume and sperm concentration were recorded, while the viability, motility, and IPM percentages were similar (Table 2).

### 3.1. The Effect of Protein Doses

After 24 h, a dramatic decline in sperm viability, progressive motility, and IPM was observed in the fresh semen samples with SM-EY extender only (T0) at 5°C, which decreased by 40.50 ± 0.36%, 37.50 ± 0.83%, and 35.70 ± 0.21%, respectively. The addition of 12 *μ*g of the 150 kDa protein/100 mL to the SM-EY extender (T1) attenuated the decline in sperm viability, progressive motility, and IPM after 24 h at 5°C, which decreased by 11.85 ± 0.71%, 9.95 ± 0.28%, and 6.85 ± 0.36%, respectively (Table 3).

The addition of 12 *μ*g of the protein significantly increased (*p* < 0.05) the sperm quality compared to the control (T0 to T1); however, the higher dose (24 *μ*g) of the protein (T1 to T2) resulted in a significant decrease in sperm quality (*p* < 0.05). Semen in the T1 treatment had the highest viability, motility, and IPM levels and the lowest MDA levels.

### 3.2. The Effect of Storage Length

Overall, from the first day (D1) to the fifth day (D5) stored at 5°C, the survival of sperm in terms of viability, progressive motility, and IPM percentages consistently decreased, and MDA concentration and sperm apoptosis percentage increased. The quality of the semen was significantly reduced (*p* < 0.05) the longer it was stored (rows in Table 4).

The use of 60-million motile sperm per dose for intracervical AI produces satisfactory pregnancy rates in Kacang goat does [14]. Based on the data presented in Tables 1 and 2, it could determine the average total motile sperm per ejaculate and the number of doses and volume (mL) necessary for successful intracervical AI (Table 5). The number of sperm in progressive motility per day of storage could be calculated with the following formula: ejaculate volume (mL) × sperm concentration (million/mL) × percentage progressive sperm motility per day of storage (Table 4). The average of doses was obtained by calculating the number of sperm in progressive motility per day of storage divided by 60 (million motile sperm). Meanwhile, the volume required for intracervical insemination (mL) was determined from the volume of extended semen (mL) divided by the number of doses per ejaculate.

## 4. Discussion

Fresh Simental bull semen was collected, and fertility was confirmed based on sperm viability and motility (≥70%) and concentration (≥600 million sperm/ml of semen) [1]. Bull seminal plasma contains several organic compounds, including IGF-I, which has a molecular weight of 7,649 Daltons [26]. IGF-I, a potent mitogenic and metabolic substance, is an important regulator of reproductive functions [27]. IGF-I in seminal plasma is primarily produced by the testicular and epididymal organs [28]. However, most local tissue IGF does not exist as free molecules [29] but forms a ternary complex that consists of IGF-I, IGFBP-3, and acid-labile subunit with a molecular weight of 150 kDa [30]. The higher molecular mass of the ternary complex prolongs the half-life of IGF-I from less than 5 min to 16 h [15].

Physiologically, sperm must maintain low ROS levels to preserve normal functions [31], such as maturation, hyperactivation, acrosome reaction, and sperm-oocyte fusion [32]. At the molecular level, ROS play a decisive role in tyrosine phosphorylation, sterol oxidation, and cholesterol efflux in the spermatozoon capacitation and fertilization processes [33]. In seminal plasma, ROS balancing is mediated by the mitochondrial metabolism and endogenous antioxidants [34]. Increased ROS production disturbs the oxidant-antioxidant balance, resulting in oxidative stress and finally damage to the sperm [35]. Therefore, an exogenous antioxidant can be added to rebalance the system during semen storage at 5°C.

The average progressive motility of Kacang goat ejaculate was 89 ± 2.80% (Table 2), which was higher than the previous report of 75.2% [2] and lower than the 94.8% reported by Setiawan et al. [36]. The ejaculates were qualified for AI based on the percentage (more than 70%) of progressive sperm motility.

In this study, Kacang goat semen viability was extended using an SM-EY extender [14]. Skim milk contains sulfhydryl, *β*-lactoglobulin, phosphocaseinate, and casein micelle, which protect the plasma membrane from lipid peroxidation [37]. Egg yolk is rich in cholesterol [38], which improves the quality of stored semen by decreasing mitochondrial deterioration and inhibiting ROS production [39].

### 4.1. Effects of the Protein Supplementation

Without protein supplementation, sperm viability, progressive motility, and IPM percentages decreased rapidly in the first 24 h after collection (T0, Table 4). The observed deterioration in sperm quality can be attributed to oxidative stress resulting from excessive ROS generation as redox reactions in the mitochondria produce adenosine triphosphate (ATP). Polyunsaturated fatty acids within the plasma membrane readily accept unpaired electrons from ROS [31], causing lipid peroxidation and subsequent MDA production. Thus, MDA concentrations reflect the level of oxidative damage to the plasma membrane [10]. High ROS levels cannot be offset by endogenous antioxidants (glutathione peroxidase, catalase, and superoxide dismutase), resulting in reduced membrane integrity, increased membrane permeability, decreased sperm viability and motility [34], and finally sperm apoptosis [40]. IGF-I enhances endothelial antioxidant activity, primarily via the upregulation of glutathione peroxidase-1 (GPX1) expression and activity [41]. Supplementation of SM-EY extender with the IGF-I complex protein resulted in higher viability, motility, and IPM and decreased MDA concentration and apoptotic sperm compared to those without protein supplementation. However, the higher dose of protein (24 *μ*g) was less effective at maintaining sperm quality than the 12 *μ*g dose (columns in Table 4). This may be because the higher exposure of antioxidants results in a paradox of antioxidants that decrease male fertility [42, 43].

Compared to semen with the SM-EY extender alone (T0), supplementation of 12 *μ*g of the protein/100 mL SM-EY extender (T1) reduced the decrease in sperm viability and progressive motility after 24 h stored at 5°C by approximately ¼, while the decrease in IPM was only 1/6 that of sperm without the protein supplementation (Table 3). The IGF-I protein complex was expected to act as an antioxidant in the SM-EY extender. Antioxidant supplementation can prevent cellular damage or death [44] and affect tyrosine phosphorylation and cholesterol efflux to improve sperm motility [45]. To fertilize the ovum, sperm require energy that is produced by the mitochondria to drive the flagella (sperm motility mechanism). The vibration of two central singlet microtubules encircled by nine outer doublet microtubules is regulated by Ca^2+^ and cyclic adenosine monophosphate (cAMP) to produce flagellar motion [34].

Supplementation of IGF-I has been demonstrated to protect sperm during the cryopreservation process, but the effects do not result from direct antioxidant activity [46]. IGF-I complex binds to its receptor on the sperm plasma membrane [47] and is thought to act indirectly as an antioxidant by increasing Ca^2+^ in the cells, decreasing cAMP, and subsequently decreasing ATP production [30]. ATP production generates ROS, which disrupt the sperm plasma membrane via lipid peroxidation [10]. Damage to the membrane structure disrupts membrane functions, resulting in an abnormal increase in the influx of Ca^2+^ into cells. The increase in Ca^2+^ concentration within the cells will activate the sperm enzymes, producing excessive fatty acids and anionic superoxide radicals. High calcium will also increase membrane permeability and activate endonuclease, which will destroy transglutaminases that covalently bind with membrane proteins through the formation of isopeptides [48]. There is a positive association between circulating IGF-I, IGFBP3, and serum calcium [49].

### 4.2. The Effect of Storage Length

In the control group, sperm were stored in the SM-EY extender only, which provides energy for life and motility. ROS are generated as a byproduct of ATP energy production [31]; the progressive accumulation of ROS with increasing storage time reduces sperm membrane integrity, viability, and motility [32]. Sperm survival decreased over five days of storage at 5°C. The viability, progressive motility, and IPM decreased, while MDA concentration and apoptosis increased. Survival time is defined as the duration from the initial semen collection until the minimum quality required for AI is reached. The viability of sperm decreases with increasing storage time due to ROS accumulation [50, 51]. Storage temperature also influences semen viability. Goat sperm survival time is five days at room temperature and only 17 h at 5°C [52]. However, sheep sperm stored at 4°C survived longer than at 23°C [9]. The storage of semen in an SM-EY extender at low temperatures was intended to extend the life span of sperm. However, the extent of storage time directly affects ROS levels. Under the conditions of ROS overproduction and chilled storage, the seminal plasma antioxidant system is insufficient to balance ROS levels over a long period [10]. ROS accumulate over time, decreasing the IPM, viability, and motility and increasing MDA levels and apoptosis (rows in Table 4). Moreover, when semen is stored with an extender, sperm metabolic processes increase, forming lactic acid and decreasing the pH of the medium. The decrease in pH disrupts the metabolic process, limiting ATP production and subsequently reducing sperm motility [53].

For goat AI, the progressive motility of extended semen must remain above 40% [1]. When stored at 5°C with SM-EY extender, sperm progressively motility was maintained ≥40% for five days in T1 and three days in T0 (control) and T2. The control group outperformed refrigerator-preserved indigenous ram semen, which maintained the minimum motility for only 48 h in skim milk extender [54]. However, the control group in this study was inferior to Ettawa goat semen in skim milk extender, which maintained the threshold of progressive motility for four days at 5°C [6].

In a previous study, a dose of 60-million motile sperm per intracervical AI resulted in a pregnancy rate of 94.73% in Kacang goats [14]. Based on the insemination dose, a single Kacang goat ejaculate could be divided into 57 doses (0.38 mL volume) with 12 *μ*g IGF-I complex protein/mL SM-EY extender (T1). Bubenickova et al. [55] reported a positive correlation between the concentration of IGF-I complex protein in horse seminal plasma and the concentration, morphology, and motility of sperm. In addition, IGF-I maintained ram sperm functions following chilled storage [56] by providing a stable energy supply for sperm metabolism [16]. The lower seminal plasma IGF-I concentration was also significantly correlated with the percentage of abnormal sperm morphology [57]. The proposed IGF-1 mechanisms for maintaining motility are through energy metabolism by increasing glucose uptake, pyruvate dehydrogenase activity, and antioxidant effects along with storage [58].

## 5. Conclusion

The addition of 12 *μ*g of the protein/mL skim milk-egg yolk extender to goat semen stored at cold temperatures was effective for maintaining the best sperm quality in terms of motility, viability, and plasma membrane integrity and inhibiting MDA production and apoptosis.

## Figures and Tables

**Table 1:** The average molecular weight (kDa) proteins, percentage or fraction, and concentration (ng/mL) of Simmental bull seminal plasma.

Band no.	Molecular weight (kDa)	Fraction (%)	Concentration (*μ*g/mL)
1	150.29 ± 0.83	4.58 ± 0.16	1.18 ± 0.04
2	95.89 ± 0.68	12.42 ± 0.24	3.20 ± 0.06
3	46.02 ± 0.36	6.02 ± 1.54	1.55 ± 0.40
4	24.82 ± 0.47	31.93 ± 4.21	8.22 ± 1.08
5	14.04 ± 0.50	9.89 ± 2.36	2.55 ± 0.61
6	11.47 ± 0.76	35.17 ± 5.99	9.05 ± 1.54

**Table 2:** Characteristics of Simmental bull and Kacang goat ejaculate.

Parameters	Bull semen	Goat semen
Volume (mL)	9.15 ± 0.20	2.2 ± 0.35
Concentration (million) of sperm (mL)	1,035 ± 236.50	3,945 ± 243
Viability (%)	92.65 ± 2.40	89 ± 2.80
Progressive motility (%)	86.80 ± 2.60	84 ± 2.75
Intact plasma membrane (%)	80.35 ± 2.10	82 ± 1.75

**Table 3:** The daily percent decrease in sperm viability, motility, and intactness of the plasma membrane of Kacang goat semen stored for five days at 5°C in skim milk-egg yolk extender with and without the addition of 150 kDa protein isolated from Simmental bull seminal plasma.

Parameters	Fresh to D1	Fresh to D2	Fresh to D3	Fresh to D4	Fresh to D5
*Viability (%)*					
T0	40.50 ± 0.36^Aa^	43.61 ± 0.61^Cb^	47.51 ± 0.51^Cb^	55.71 ± 0.71^Bb^	62.61 ± 0.61^Bb^
TI	11.85 ± 0.71^Bc^	13.56 ± 0.56^Cc^	24.41 ± 0.41^Ba^	34.61 ± 0.61^Ba^	54.71 ± 0.71^Aa^
TII	29.90 ± 0.76^Ab^	38.66 ± 0.66^Ba^	43.46 ± 0.46^Cb^	50.76 ± 0.76^Bb^	57.61 ± 0.61^Bb^

*Motility (%)*					
T0	37.50 ± 0.83^Aa^	41.08 ± 0.08^Bb^	44.08 ± 0.08^Bc^	53.88 ± 0.88^Ba^	60.98 ± 0.98^Bb^
TI	9.95 ± 0.28^Bc^	10.93 ± 0.93^Cc^	21.88 ± 0.88^Ba^	32.08 ± 0.08^Ba^	44.28 ± 0.28^Aa^
TII	26.90 ± 0.23^Ab^	36.13 ± 0.13^Ba^	40.83 ± 0.83^Db^	48.23 ± 0.23^Cb^	56.08 ± 0.08^Cb^

*IPM (%)*					
T0	35.70 ± 0.21^Aa^	41.36 ± 0.36^Cb^	42.36 ± 0.36^Dc^	50.26 ± 0.26^Bb^	58.26 ± 0.26^Ba^
TI	6.85 ± 0.36^Ac^	12.21 ± 0.21^Db^	20.36 ± 0.36^Ba^	29.26 ± 0.26^Ba^	36.36 ± 0.36^Cb^
TII	28.90 ± 0.41^Ab^	36.31 ± 0.31^Ba^	38.21 ± 0.21^Cb^	45.41 ± 0.41^Bb^	52.41 ± 0.41^Bb^

Different capital letter superscripts in the same row and lowercase letter superscripts in the same column indicate significant differences (*p* < 0.05). SM-EY: skim milk-egg yolk; T0: SM-EY extender without the protein; T1: SM-EY extender +12 *μ*g protein/100 mL extender; T2: SM-EY extender +24 *μ*g protein/100 mL extender.

**Table 4:** The average viability, motility, intactness of plasma membrane percentages, MDA concentration, and percentage of apoptotic sperm of Kacang goat sperm stored at 5°C for five days in skim milk-egg yolk extender with and without the addition of 150 kDa protein isolated from Simmental bull seminal plasma.

Parameters	Day 1	Day 2	Day 3	Day 4	Day 5
*Viability (%)*					
T0	48.50 ± 0.40^Ac^	45.25 ± 0.05^Bc^	41.35 ± 0.05^Cc^	33.15 ± 0.05^Dc^	26.25 ± 0.20^Ec^
TI	**77.15** **±** **0.25**^**Aa**^	**75.30** **±** **0.25**^**Ba**^	**64.45** **±** **0.55**^**Ca**^	**54.25** **±** **0.35**^**Da**^	**34.15** **±** **0.15**^**Ea**^
TII	59.10 ± 0.25^Ab^	50.20 ± 0.25^Bb^	45.40 ± 0.35^Cb^	38.10 ± 0.45^Db^	31.25 ± 0.40^Eb^

*Motility (%)*					
T0	46.50 ± 0.40^Ac^	43.25 ± 0.25^Bc^	40.25 ± 0.05^Cc^	30.45 ± 0.05^Dc^	23.35 ± 0.10^Ec^
TI	**74.05** **±** **0.35**^**Aa**^	**73.40** **±** **0.05**^**Ba**^	**62.45** **±** **0.85**^**Ca**^	**52.25** **±** **0.40**^**Da**^	**40.05** **±** **0.15**^**Ea**^
TII	57.10 ± 0.35^Ab^	48.20 ± 0.35^Bb^	43.50 ± 0.75^Cb^	36.10 ± 0.45^Db^	28.25 ± 0.75^Eb^
IPM (%)					
T0	46.30 ± 0.30^Ac^	41.15 ± 0.05^Bc^	40.15 ± 0.05^Cc^	32.25 ± 0.05^Dc^	24.25^c^ ± 0.20^Ec^
TI	**75.15** **±** **0.25**^**Aa**^	**70.30** **±** **0.25**^**Ba**^	**62.15** **±** **0.25**^**Ca**^	**53.25** **±** **0.35**^**Da**^	**46.15** ^**a**^ **± 0.15** ^**Ea**^
TII	53.10 ± 0.25^Ab^	46.20 ± 0.25^Bb^	44.30 ± 0.15^Cb^	37.10 ± 0.25^Db^	30.10^b^ ± 0.30^Eb^

*MDA (ng/mL)*					
T0	2088.37 ± 35.18^Aa^	2216.32 ± 37.70^Ba^	2455.26 ± 37.75^Ca^	2705.59 ± 50.75^Da^	2980.50 ± 22.55^Ea^
TI	**1556.35** **±** **40.20**^**Ac**^	**1666.19** **±** **33.20**^**Bc**^	**1835.35** **±** **25.19**^**Cc**^	**1976.54** **±** **34.23**^**Dc**^	**2106.54** **±** **30.35**^**Ea**^
TII	1845.20 ± 23.10^Ab^	2052.28 ± 30.15^Bb^	2150.73 ± 35.80^Cb^	2530.42 ± 46.12^Db^	2580.40 ± 12.10^Eb^

*Apoptosis*					
T0	4.25 ± 0.15^Ca^	5.25 ± 0.55^Ba^	5.75 ± 0.07^Ba^	6.15 ± 0.05^Aa^	6.25 ± 0.10^Aa^
TI	**2.40** **±** **0.35**^**Cc**^	**2.70** **±** **0.25**^**Cc**^	**3.35** **±** **0.45**^**Bc**^	**3.60** **±** **0.25**^**Bc**^	**4.15** **±** **0.15**^**Ac**^
TII	3.70 ± 0.35^Cb^	4.30 ± 0.15^Bb^	5.05 ± 0.20^Ab^	5.40 ± 0.15^Ab^	5.50 ± 0.25^Ab^

Different capital letter superscripts in the same row and lowercase letter superscripts in the same column indicate significant differences (*p* < 0.05). SM-EY: skim milk-egg yolk; T0: SM-EY extender without the protein; T1: SM-EY extender +12 *μ*g protein/100 mL extender; T2: SM-EY extender +24 *μ*g protein/100 mL extender. The parameters of the best quality semen are in bold. Values above the minimum progressive sperm motility (40%) for intracervical artificial insemination are indicated by italics.

**Table 5:** The number of motile sperm per ejaculate, average number of doses, and volume (mL) per dose for intracervical insemination using Kacang goat semen stored at 5°C for five days in skim milk-egg yolk extender with and without the addition of 150 kDa protein isolated from Simmental bull seminal plasma. 5°C for five days.

Group	Day 1	Day 2	Day 3	Day 4	Day 5
*T0*	**3975.42** **±** **39.95**	**3697.57** **±** **24.97**	**3441.09** **±** **4.99**	**2603.26** **±** **4.99**	**1996.26** **±** **9.99**
	(66/0.32)	(62/0.35)	(57/0.37)	(43/0.49)	(33/0.64)

*T1*	**6330.76** **±** **34.96**	**6275.19** **±** **4.99**	**5339.04** **±** **84.89**	**4467.01** **±** **39.95**	**3423.99** **±** **14.98**
	(106/0.20)	(105/0.20)	(89/0.24)	(74/0.29)	(57/0.38)

*T2*	**4881.65** **±** **34.96**	**4120.76** **±** **74.91**	**3718.95** **±** **74.91**	**3086.30** **±** **44.94**	**2415.18** **±** **74.91**
	(81/0.26)	(69/0.31)	(62/0.35)	(51/0.42)	(40/0.53)

The number of motile sperm per ejaculate is shown in bold; the number of doses with 60-million motile sperm cells for intracervical insemination/volume (mL) is in brackets. SM-EY: skim milk-egg yolk; T0: SM-EY extender without the protein; T1: SM-EY extender +12 *μ*g protein/100 mL extender; T2: SM-EY extender +24 *μ*g protein/100 mL extender.

## Data Availability

The data of this study are available from the corresponding author upon request.

## References

[1] INSA (Indonesian National Standard Agency) (2014). Frozen semen–part 3: goat and sheep. http://bibit.ditjenpkh.pertanian.go.id/sites/.

[2] Kusumawati E. D., Utomo K. N., Krisnaningsih A. T. N., Rahadi S. (2017). Kualitas semen kambing kacang dengan Lama simpan yang berbeda pada suhu ruang menggunakan tris aminomethan kuning telur. *Jurnal Ilmu dan Teknologi Peternakan Tropis*.

[3] Am-In N., Tantasuparuk W., Manjarin R., Kirkwood R. N. (2011). Effect of site of sperm deposition on fertility when sows are inseminated with aged semen. *Journal of Swine Health and Production*.

[4] Macías A., Ferrer L. M., Ramos J. J. (2017). Technical Note: a new device for cervical insemination of sheep - design and field test1. *Journal of Animal Science*.

[5] Di Iorio M., Manchisi A., Rocco M., Chrenek P., Iaffaldano N. (2014). Comparison of different extenders on the preservability of rabbit semen stored at 5°C for 72 hours. *Italian Journal of Animal Science*.

[6] Susilowati S., Triana I. N., Wurlina W., Arimbi A., Srianto P., Mustofa I. (2019). Addition of L-arginine in skim milk extender maintains goat spermatozoa quality in chilled temperature for five days. *Veterinary World*.

[7] Susilowati S., Mustofa I., Wurlina W., Hernawati T., Oktanella Y. (2021). Green tea extract was expand the threshold period of goat sperm in chilled storage. *Journal of Animal Science*.

[8] Korkmaz M. K., Emsen E., Köker A., Kocamüftüolu M. (2017). Duration effect of fresh semen kept in vitro on sheep conception rate. *Animal Reproduction*.

[9] Wusiman A., Wang Y.-P., Ren K. (2012). Semen storage at 23, 4 or −196°C and its application to artificial insemination in small-tail han sheep. *Asian Journal of Animal and Veterinary Advances*.

[10] Wen F., Li Y., Feng T. (2019). Grape seed procyanidin extract (GSPE) improves goat sperm quality when preserved at 4°C. *Animals*.

[11] Saraswati S., Jindal S. K., Kharche S. D. (2016). Antioxidant and sperm: a complex story-a Review. *Indian Journal of Animal Sciences*.

[12] Saha S., Dungdung S., Majumder G. (2014). Determination of the antioxidant potential of goat sperm cells. *Oxidants and Antioxidants in Medical Science*.

[13] Susilowati S., Triana I. N., Suprayogi T. W., Arimbi A., Wurlina W. (2018). Effect of bovine seminal protein on the quality of frozen sperm from goats. *Philippine Journal of Veterinary Medicine*.

[14] Susilowati S., Triana I. N., Sardjito T., Suprayogi T. W., Wurlina W., Mustofa I. (2020). Effect of Simmental bull seminal plasma protein in egg yolk-citrate extender on Kacang buck semen fertility. *Cryobiology*.

[15] Allard J. B., Duan C. (2018). IGF-binding proteins: why do they exist and why are there so many?. *Frontiers in Endocrinology*.

[16] Selvaraju S., Sivasubramani T., Raghavendra B. S. (2012). Effect of dietary energy on seminal plasma insulin-like growth factor-I (IGF-I), serum IGF-I and testosterone levels, semen quality and fertility in adult rams. *Theriogenology*.

[17] Aulanni’am A. (2005). *Proteins and Its Analysis*.

[18] Kurien B. T., Scofield R. H. (2015). Western blotting: methods and protocols. *Methods in Molecular Biology*.

[19] Mishra M., Tiwari S., Gomes A. V. (2017). Protein purification and analysis: next generation Western blotting techniques. *Expert Review of Proteomics*.

[20] Cima-Cabal M. D., Vazquez F., de Los Toyos J. R., del Mar García-Suárez M. (2019). Protein expression analysis by western blot and protein-protein interactions. *Methods in Molecular Biology*.

[21] Bass J. J., Wilkinson D. J., Rankin D. (2017). An overview of technical considerations for Western blotting applications to physiological research. *Scandinavian Journal of Medicine & Science in Sports*.

[22] Mohammadian T., Doosti M., Paknejad M., Siavoshi F., Massarrat S. (2010). Preparative SDS-PAGE electroelution for rapid purification of alkyl hydroperoxide reductase from *Helicobacter pylori*. *Iranian Journal of Public Health*.

[23] Avan A., Demirci Çekiç S., Uzunboy S., Apak R. (2016). Spectrophotometric determination of phenolic antioxidants in the presence of thiols and proteins. *International Journal of Molecular Sciences*.

[24] Mustofa I., Susilowati S., Wurlina W., Hernawati T., Oktanella Y. (2021). Green tea extract increases the quality and reduced DNA mutation of post-thawed Kacang buck sperm. *Heliyon*.

[25] Susilowati S., Mustofa I., Wurlina W., Triana I. T., Utama S., Rimayanti R. (2021). Effect of insulin-like growth factor-1 complex of simmental bull seminal plasma on post-thawed kacang buck semen fertility. *Veterinary World*.

[26] Velho A. L. C., Menezes E., Dinh T. (2018). Metabolomic markers of fertility in bull seminal plasma. *PLoS One*.

[27] Lewitt M. S., Boyd G. W. (2019 Apr 17). The role of insulin-like growth factors and insulin-like growth factor-binding proteins in the nervous system. *Biochemistry Insights*.

[28] Mora Rodríguez J. A., Porchia L. M., Camargo F., López-Bayghen E. (2019). The use of insulin-like growth factor 1 improved the parameters of the seminogram in a patient with severe oligoasthenoteratozoospermia. *SAGE Open Medical Case Reports*.

[29] Bach L. A. (2018). 40 years OF IGF1: IGF-binding proteins. *Journal of Molecular Endocrinology*.

[30] Beigi Harchegani A., Irandoost A., Mirnamniha M., Rahmani H., Tahmasbpour E., Shahriary A. (2019). Possible mechanisms for the effects of calcium deficiency on male infertility. *International Journal of Fertility and Sterility*.

[31] Wagner H., Cheng J. W., Ko E. Y. (2018). Role of reactive oxygen species in male infertility: an updated review of literature. *Arab Journal of Urology*.

[32] Turk G. (2015). Physiological and pathological effects of reactive oxygen species on spermatozoon functions. *Turkiye Klinikleri*.

[33] Takeshima T., Kuroda S., Yumura Y. (2018). Reactive oxygen species and sperm cells.

[34] Pereira R., Sá R., Barros A., Sousa M. (2015). Major regulatory mechanisms involved in sperm motility. *Asian Journal of Andrology*.

[35] Thompson A., Agarwal A., Du Plessis S. S., Parekattil S. J., Agarwal A. (2014). The physiological role of reactive oxygen species in sperm function: a review. *Antioxidants in Male Infertility: A Guide for Clinicians and Researchers*.

[36] Setiawan F., Kusumawati E. D. (2017). The quality of fresh semen Kacang Goat at 5° C storage with and without an extender. *Journal of Animal Science*.

[37] Fadl A. M., Ghallab A. M., Abou-Ahmed M. M. (2020). Comparison between Tris-buffer and INRA-82 extenders on the quality of chilled rabbit spermatozoa. *World Rabbit Science*.

[38] Anzar M., Rajapaksha K., Boswall L. (2019). Egg yolk-free cryopreservation of bull semen. *PLoS One*.

[39] Ezz M. A., Montasser A. E., Hussein M. (2017). The effect of cholesterol loaded cyclodextrins on post-thawing quality of buffalo semen in relation to sperm DNA damage and ultrastructure. *Reproductive Biology*.

[40] Manente L., Pecoraro S., Picillo E. (2015). Molecular evidence of apoptotic pathway activation in semen samples with high DNA fragmentation. *In Vivo*.

[41] Higashi Y., Pandey A., Goodwin B., Delafontaine P. (2013). Insulin-like growth factor-1 regulates glutathione peroxidase expression and activity in vascular endothelial cells: implications for atheroprotective actions of insulin-like growth factor-1. *Biochimica et Biophysica Acta-Molecular Basis of Disease*.

[42] Majzoub A., Agarwal A., Esteves S. C. (2017). Antioxidants for elevated sperm DNA fragmentation: a mini review. *Translational Andrology and Urology*.

[43] Majzoub A., Agarwal A. (2018). Systematic review of antioxidant types and doses in male infertility: benefits on semen parameters, advanced sperm function, assisted reproduction and live-birth rate. *Arab Journal of Urology*.

[44] El-Battawy K. (2019). Preservation of goat semen at 5°C with emphasis on its freezability and the impact of melatonin. *International Journal of Veterinary Sciences Research*.

[45] Martin-Hidalgo D., Bragado M. J., Batista A. R., Oliveira P. F., Alves M. G. (2019). Antioxidants and male fertility: from molecular studies to clinical evidence. *Antioxidants*.

[46] Kumar P., Suman, Pawaria S. (2019). Serum and seminal plasma IGF-1 associations with semen variables and effect of IGF-1 supplementation on semen freezing capacity in buffalo bulls. *Animal Reproduction Science*.

[47] Ipsa E., Cruzat V. F., Kagize J. N., Yovich J. L., Keane K. N. (2019). Growth hormone and insulin-like growth factor Action in reproductive tissues. *Frontiers in Endocrinology*.

[48] Jin S.-K., Yang W.-X. (2017). Factors and pathways involved in capacitation: how are they regulated?. *Oncotarget*.

[49] Van Hemelrijck M., Shanmugalingam T., Bosco C., Wulaningsih W., Rohrmann S. (2015). The association between circulating IGF1, IGFBP3, and calcium: results from NHANES III. *Endocrine Connections*.

[50] Hahn K., Failing K., Wehrend A. (2019). Effect of temperature and time after collection on buck sperm quality. *BMC Veterinary Research*.

[51] Agarwal A., Virk G., Ong C., du Plessis S. S. (2014). Effect of oxidative stress on male reproduction. *The World Journal of Men’s Health*.

[52] Ferdinand N., Thomas T. T., Augustave K., Henry D. F., Fernand T., Etienne P. T. (2012). Effects of goat age, storage duration, storage temperature and diluent on fresh west african dwarf goat semen. *Journal of Reproduction and Infertility*.

[53] Matsuzaki M., Mizushima S., Hiyama G. (2015). Lactic acid is a sperm motility inactivation factor in the sperm storage tubules. *Scientific Reports*.

[54] Rahman M., Gofur M., Rahman M., Bari F., Juyena N. (2018). Effect of skim milk and tris-citrate extenders to preserve the semen of indigenous ram of Bangladesh. *Asian Journal of Biology*.

[55] Bubenickova F., Postlerova P., Simonik O., Sirohi J., Sichtar J. (2020). Effect of seminal plasma protein fractions on stallion sperm cryopreservation. *International Journal of Molecular Sciences*.

[56] Makarevich A. V., Spalekova E., Olexikova L., Kubovicova E., Hegedusova Z. (2014). Effect of insulin-like growth factor I on functional parameters of ram cooled-stored spermatozoa. *Zygote*.

[57] Selvaraju S., Subramani T., Raghavendra B. S., Ravindra J. P. (2010). Effect of IGF-I on spermatozoa membrane protein profile and correlation between seminal plasma IGF-I and antioxidant enzymes in buffalo semen. *The Indian Journal of Animal Sciences*.

[58] Poudel S. B., Dixit M., Neginskaya M. (2020). Effects of GH/IGF on the aging mitochondria. *Cells*.

